# Supplementary material for: The genetic characterization of fall armyworm populations in Ecuador and its implications to migration and pest management in the northern regions of South America
Source: PLoS One. 2020 Aug 3;15(8):e0236759. doi: 10.1371/journal.pone.0236759 (PMC7398513; doi:10.1371/journal.pone.0236759)
Supplement: Attachment — Submitted filename: Response to Reviewer.docx [file pone.0236759.s001.docx]

Reviewer comments in <italics>:

< *To help the reader understand the results and implications of this study, the authors should provide some additional detail on the fall armyworm collections. In particular:
• How were collection locations in Ecuador chosen? Do they correspond to major cropping areas? Are they separated by significant physical barriers?*>

The description of sampling in Ecuador was expanded to include how sites were chosen and now mentioning that they were at major cropping areas (lines 118-121). We now mention the Andes Mountains as a potential significant barrier (lines 103-106) and indicate the location of the Andes relative to the collection sites in Figure 1.

We also reorganized the data to now directly address the possibility of the Andes acting as a physical barrier by grouping the collections by region. This is shown in Table 2 and Fig 6 and discussed in lines 371-381.

<• *When were collections made (date)? Do they span some period of time or were they largely synchronous*?>

The timing of the collections is described in lines 121-125 and are listed in Table 1.
